# The Contagion of Scarlet Fever

**Published:** 1875-05

**Authors:** 


### THE CONTAGION OF SCARLET FEVER.

None of the diseases common in this country is more readily communicated by contagion than scarlet fever. It is probable that no other contagion retains for so long a period its power of producing disease; and certain it is that none can be more insidious in its movements and operations. It attaches itself to the clothing of persons in the infected room; it lurks in the carpet and upholstery; it may conceal itself among articles of clothing packed in trunks and drawers, and come forth after many weeks with its disease-producing powers active and unimpaired.

It is impossible to say how long the contagious poison, when thus confined, will retain its power. There is no doubt that it may be carried for a considerable time in clothing worn upon the person. Woolen shawls, cloaks and furs afford excellent means of transit for the contagion. Persons wearing such articles should be especially careful not to pass from a scarlet fever patient to the presence of children who are liable to take the disease. Clean clothes from the washerwoman, and new garments from the tailor may be the means of bearing contagion. It is probable that scarlet fever and other diseases, more or less prevalent in our cities, are frequently communicated in this way.

During what period in the course of scarlatina can the patient "give" the disease? Certainly from the date of the appearance of the eruption, and perhaps from the commencement of the fever, until—when? It has been said that the completion of the process of "peeling" or scurfing marks the end of the period of personal infection. The scurf skin is highly charged with the matter of contagion, and the minute particles are so freely thrown off that they may be detected floating in the air of the room. Then it may be said, without exaggeration, that the atmosphere of the department is thick with poison. Recent observation has demonstrated that scarlatina is communicated long after the skin has finished peeling and is apparently smooth and sound. Even until the eighth or ninth week from the height of the disease, infection from the person may take place. The old idea of quarantine, with all the *forty days* that the term implies, expresses very nearly the length of time that a scarlatina patient should be excluded from the healthy. The communication of the disease in the eighth or ninth week may be regarded as exceptional.

The period of incubation, or the time from the exposure to contagion to the development of the fever, may be stated as from two to six days. If a person remains unaffected for a week after exposure, he may be regarded as having escaped.

Since none of our common diseases is more dreaded than scarlet fever, it is important to know what can be done to prevent its diffusion. To this end the following conditions should be observed: Let the patient be assigned an upper chamber, freely ventilated, comfortably warm and reasonably cool. There should be no carpet on the floor, no curtains about the bed, and no cushioned and upholstered chairs or sofas. Use an iron bedstead, and if possible let the bedding be destroyed by fire as soon as the patient is so far recovered as to mingle with the other members of the family. The handkerchiefs which he has used should be burned. It is better to furnish bits of old cotton or linen in place of handkerchiefs, and each piece as soon as soiled may be removed and destroyed. The spittoon, or other vessel which receives the expectoration, should contain a little water with carbolic acid or chloride of lime. One or the other of these disinfectants must be used freely about the room. An indispensable article for the comfort of the patient is an earth-closet. There are several kinds in use, but as far as we have observed the simplest and cheapest is the best. Common garden soil, perfectly dry, should be used, or sifted coal-ashes. Either of these substances will readily deodorise the intestinal discharges. A small quantity of sulphate of iron—coperas—if added to the soil or ashes, will serve as an excellent disinfectant.

Nurses and others who come in contact with the patient, should frequently wash their hands with carbolic soap. Clothing taken from the patient, if not burned, should be allowed to remain in water containing one of the above named disinfectants, two or three days before being washed.

These simple suggestions, if followed, would result in a greatly decreased number of cases of scarlet fever ; and if the means proposed were generally adopted, we believe that in a short time the disease would be effectually "stamped out."

We are aware that in many families the course we propose is impracticable. They have not the room, nor the means. But they can follow some of our directions. They can ventilate the sick-room, and use disinfectants.

It is possible that physicians, in treating this disease, neglect, sometimes, an important part of their duty. They do not enforce, as they ought, the simple regulations by which so much may be done toward destroying the *contagion.*

---

Exercise, air and sunlight are three of nature's most potent remedies.

